# ITGA2B基因复合杂合突变所致遗传性血小板无力症家系分析及致病机制研究

**DOI:** 10.3760/cma.j.cn121090-20230816-00070

**Published:** 2024-04

**Authors:** 晓梅 卢, 栋彦 付, 耀方 张, 丽东 赵, 蕾 王, 嘉 杨, 杰 刘, 嘉伟 郑, 林花 杨, 刚 王

**Affiliations:** 山西医科大学第二临床医学院（第二医院）血液科、山西医科大学血液病学研究所，太原 030001 Institute of Hematology: The Second Hospital of Shanxi Medical University, Taiyuan 030001, China

**Keywords:** 遗传性血小板无力症, ITGA2B基因, 整合素αⅡbβ3, mRNA剪接, 无义突变, Hereditary Glanzmann thrombasthenia, ITGA2B Gene, Integrin αⅡbβ3, mRNA Splicing, Nonsense mutation

## Abstract

**目的:**

对一个ITGA2B基因复合杂合突变导致的遗传性血小板无力症家系进行表型及基因型研究，并探索其分子致病机制。

**方法:**

使用二磷酸腺苷、胶原、肾上腺素、花生四烯酸及瑞斯托霉素等诱聚剂进行血小板聚集试验，检测先证者及家系成员的血小板聚集率。通过流式细胞术检测血小板表面CD41（αⅡb）、CD61（β3）、CD42b（GPⅠb）的表达。采用基因测序技术进行基因鉴定。利用RT-PCR检测ITGA2B基因mRNA剪接情况，qRT-PCR检测ITGA2B基因mRNA相对水平。生物信息学分析评估突变位点的致病性及对蛋白结构和功能的影响。通过Western blot检测分析血小板总αⅡb、β3的表达。

**结果:**

除瑞斯托霉素外其他4种诱聚剂均无法使先证者血小板聚集。流式细胞术检测先证者血小板表面αⅡb的表达仅为0.25％，β3弱表达为9.76％，而GPⅠb表达相对正常，其余家系成员膜糖蛋白表达基本正常。基因测序结果显示先证者存在ITGA2B基因c.480C>G与c.2929C>T复合杂合突变，其中c.480C>G突变遗传自其母亲，c.2929C>T遗传自其父亲。RT-PCR及测序结果表明c.480C>G突变导致先证者及其母亲发生c.476G-574A（p.S160-S192）共99个碱基缺失的mRNA剪接。qRT-PCR检测发现c.2929C>T突变导致先证者及其父亲ITGA2B基因mRNA水平减低。生物信息学分析提示c.480C>G突变形成了与hnRNP A1蛋白结合序列，产生了5′SS剪接位点。αⅡb亚基的蛋白三维结构模型显示，p.S160-S192缺失的β-propeller结构域第2 blade缺失两条β链和一个α螺旋；c.2929C>T无义突变使得翻译提前终止产生p.R977-E1039缺失的截短型蛋白，包括胞质域（CD）、跨膜域（TM）以及胞外Calf-2结构域一条β链的缺失。Western blot检测先证者血小板总αⅡb表达缺失、β3的相对表达量为正常人的11.36％。

**结论:**

ITGA2B基因第4外显子c.480C>G与第28外显子c.2929C>T的复合杂合突变是本家系遗传性血小板无力症的致病原因。

## Abstract (en)

**Objective:**

The phenotype and genotype of a pedigree with Glanzmann thrombasthenia caused by compound heterozygous mutation in the ITGA2B gene and its molecular pathogenesis were explored.

**Methods:**

The platelet aggregation rate of the proband and his family was detected by using a platelet aggregation test with adenosine diphosphate, collagen, epinephrine, arachidonic acid, and ristocetin. The expression levels of CD41（αⅡb）, CD61（β3）, and CD42b（GPⅠb）on the platelet surface was detected by flow cytometry. Gene sequencing technology was used for the genetic identification of the family. RT-PCR was used in the detection of mRNA splicing, and qRT-PCR was used in detecting the relative mRNA level of the ITGA2B gene. Bioinformatics analysis was used to evaluate the pathogenicity of mutation sites and their effects on protein structure and function. The expressions of total αⅡb and β3 in platelets were analyzed by Western blot.

**Results:**

Except ristocetin, the other four inducers could not induce platelet aggregation in the proband. Flow cytometry showed that the expression levels of αⅡb and β3 were only 0.25％ and 9.76％, respectively, on the platelet surface of the proband, whereas GPⅠb expression was relatively normal. The expression levels of glycoproteins in the other family members were almost normal. c.480C>G and c.2929C>T mutations were detected in the proband through gene sequencing. The c.480C>G mutation was inherited from his mother, and the c.2929C>T mutation was inherited from his father. The RT-PCR and sequencing results showed that the c.480C>G mutation caused mRNA splicing in the proband and his mother, resulting in the deletion of 99 bases in c.476G-574A（p.S160-S192）. qRT-PCR showed that the c.2929C>T variant reduced the mRNA level of the ITGA2B gene in the proband and his father. Bioinformatics analysis suggested that the c.480C>G mutation might form a binding sequence with hnRNP A1 protein and generate the 5′SS splice site. The three-dimensional structural model of the αⅡb subunit showed that the β-propeller domain of the p.S160-S192 deletion lost two β-strands and one α-helix in blade 2. The c.2929C>T nonsense mutation caused premature translation termination and produced a truncated protein with the deletion of p.R977-E1039, including the cytoplasmic domain, transmembrane domain, and a β chain of the extracellular Calf-2 domain. The total αⅡb expression of the proband was absent, and the relative expression of β3 was 11.36％ of the normal level.

**Conclusion:**

The compound heterozygous mutation c.480C>G in exon 4 and c.2929C>T in exon 28 of the ITGA2B gene probably underlies Glanzmann thrombasthenia in this pedigree.

血小板无力症（Glanzmann thrombasthenia, GT）是一种罕见的常染色体隐性遗传性出血性疾病，由血小板表面整合素αⅡbβ3（又称膜糖蛋白Ⅱb/Ⅲa, GPⅡb/Ⅲa）的数量或质量缺陷引起[1]–[2]。GT的典型表现为血小板对多种生理性诱聚剂如二磷酸腺苷、胶原、肾上腺素、凝血酶等反应低下或缺如，αⅡbβ3与其主要配体纤维蛋白原结合受阻，导致血小板聚集障碍和血凝块回缩不良，但血小板计数及瑞斯托霉素诱导血小板的聚集通常正常[3]–[4]。根据血小板表面αⅡbβ3的表达情况和功能，可将GT分为3型：αⅡbβ3表达量低于正常水平的5％为Ⅰ型，正常水平的5％～20％为Ⅱ型，表达量正常但有功能缺陷的为Ⅲ型（亦称为变异型）[5]。GT的发病率估计为1/100万，以Ⅰ型最常见，Ⅲ型最少见，在近亲婚配的族群中发病率较高[6]。GT的出血可以表现为自发性或轻微创伤后的皮肤黏膜出血、鼻出血、齿龈出血、月经量增多、消化道出血、颅内出血等[6]–[7]。

αⅡb亚基和β3亚基分别由17号染色体长臂上的ITGA2B和ITGB3基因编码，其中任一基因突变都可引起GT[8]。目前GT数据库（https://glanzmann.mcw.edu/）已登记超400种ITGA2B和ITGB3基因突变，包括错义突变、无义突变、小片段缺失、插入和倒位等。不同的突变可能影响αⅡbβ3复合物形成、成熟、运输及功能，从而引起血小板表面αⅡbβ3的表达量减少或质量异常[8]–[9]。

本研究对1例ITGA2B基因复合杂合突变所致的遗传性GT家系进行表型和基因型分析并探索其分子致病机制。

## 对象与方法

一、研究对象

先证者为22岁男性，自3岁起频繁出现自发性鼻出血、齿龈出血、全身瘀点瘀斑等症状，一直未予明确诊断。2021年11月于山西医科大学第二医院血液内科门诊就诊，检测PT、APTT正常，凝血因子活性及血管性血友病因子（vWF）抗原正常，血小板计数正常，但血小板功能检测提示血小板聚集障碍。先证者父母为非近亲婚配且均无自发性出血史。本研究经山西医科大学第二医院伦理委员会批准（批件号：2023YX第159），所有受试者均已签署知情同意书。

二、试剂与仪器

二磷酸腺苷、胶原、肾上腺素、花生四烯酸及瑞斯托霉素购于美国Helena公司；CD41-FITC、CD61-PE、CD42b-PE抗体及IgG1（mouse）-FITC、IgG1（mouse）-PE同型抗体购于美国Beckman Coulter公司；D3396 Tissue DNA提取试剂盒、D2500 Gel Extraction胶回收纯化试剂盒购于美国OMEGA公司；PCR试剂TaKaRa LA Taq^®^ with GC Buffer、RNA提取试剂、逆转录试剂PrimeScript™ RT Master Mix、实时荧光定量PCR（qRT-PCR）试剂TB Green^®^ Premix Ex Taq™ II均购于日本Takara公司；哺乳动物活性蛋白抽提试剂购于上海碧云天生物技术公司；兔抗人CD41抗体、CD61抗体、β-actin抗体及HRP标记的羊抗兔IgG抗体分别购于英国abcam公司、中国沈阳万类生物、北京博奥森生物技术有限公司及英国abcam公司；超敏ECL化学发光检测试剂盒购于赛文创新（北京）生物科技有限公司；引物序列合成、Sanger测序均由北京六合华大基因科技有限公司完成；AggRAM血小板聚集仪购于美国Helena公司；Navios流式细胞仪购于美国Beckman Coulter公司；HEMA9600基因扩增仪购于珠海黑马医学仪器有限公司；7500型实时荧光定量PCR仪购于美国ABI公司。

三、血小板聚集试验

采用枸橼酸钠抗凝的外周血，200×*g*离心10 min获得富血小板血浆（Platelet-rich Plasma, PRP），1 500×*g*离心15 min获得贫血小板血浆（PPP）。分别采用二磷酸腺苷（终浓度5 µmol/L）、胶原（终浓度2.5 mg/L）、肾上腺素（终浓度75 µmol/L）、花生四烯酸（终浓度250 mg/L）、瑞斯托霉素（终浓度1.5 g/L）作为诱聚剂与PRP进行孵育，以PPP调节零点，在AggRAM血小板聚集仪上通过光学比浊法检测并绘制5 min内血小板聚集率曲线。

四、流式细胞术检测

采用EDTA抗凝的外周血，200×*g*离心5 min获得PRP，加入生理盐水1 500×*g*离心5 min，洗涤2次，制成洗涤血小板，分别使用CD41-FITC、CD61-PE、CD42b-PE抗体进行孵育，以IgG1（mouse）-FITC、IgG1（mouse）-PE抗体孵育作为同型对照，在Navios流式细胞仪上检测血小板表面CD41（αⅡb）、CD61（β3）、CD42b（GPⅠb）的表达，使用FlowJo v10.8.1软件对数据进行分析。

五、基因鉴定与分析

先证者外周血由上海派森诺生物科技有限公司进行高通量全外显子测序，获得可疑突变位点。提取该家系外周血基因组DNA，针对可疑突变位点，根据NCBI数据库（https://www.ncbi.nlm.nih.gov/）提供的ITGA2B基因参考序列（Gene ID: 3674; NG_008331.1），设计引物A2B01、A2B02（表1），按照TaKaRa LA Taq^®^ with GC Buffer说明书进行PCR扩增。PCR扩增产物由北京六合华大基因科技有限公司纯化和Sanger测序。测序结果使用SnapGene v6.0.2 软件与ITGA2B基因参考序列进行比对分析。

**表1:** PCR扩增引物序列及产物大小

引物名称	正向引物序列（5′→3′）	反向引物序列（5′→3′）	产物大小（bp）
A2B01	GTGATGAGACCCGAAATGTAGGC	CGAGGCCAGATCCAAAGCAAG	476
A2B02	ATTGCAGTGGGATTAGGTCAGAGG	ACCAGATTGGAATGGCCCTCTC	458
A2B03	GTGATGAGACCCGAAATGTAGGC	AAGAAATAATAGCCGCCAGGAGC	358/259
A2B04	GGCAGCAGAAGAAGGTGAGAG	CAGGATGTAGAGCAGGTCGG	166
β-actin	TCATCACCATTGGCAATGAG	CACTGTGTTGGCGTACAGGT	155

六、mRNA剪接及水平分析

采用枸橼酸钠抗凝的外周血，提取总RNA，逆转录合成cDNA，设计引物A2B03（表1）对cDNA进行PCR扩增，检测ITGA2B基因mRNA剪接情况。PCR产物胶回收纯化后送北京六合华大基因科技有限公司测序。设计引物A2B04（表1），按照TB Green^®^ Premix Ex Taq™ II试剂盒说明书进行qRT-PCR，以β-actin作为内参，采用ΔΔCT法分析ITGA2B基因mRNA的相对水平。

七、生物信息学分析

采用Mutation Taster（https://www.mutationtaster.org/）评估突变位点的致病性。使用ESE Finder 3.0（https://esefinder.ahc.umn.edu/）对剪接元件与剪接位点进行预测与分析。应用SWISS-MODLE（https://swissmodel.expasy.org/）对野生型和突变型蛋白三维结构进行同源建模，使用Pymol v2.6软件进行蛋白三维结构可视化编辑与分析。

八、Western blot检测蛋白表达

采用枸橼酸钠抗凝的外周血，200×*g*离心10 min获得PRP，将PRP转移到新的离心管，1 500×*g*离心10 min弃上清，沉淀为血小板，加入PBS重悬1 500×*g*离心5 min，清洗1遍，弃上清，加入适量裂解液（活性蛋白抽提试剂∶PMSF＝100∶1）提取血小板总蛋白，Western blot采用一抗兔抗人CD41、CD61及β-actin抗体、二抗羊抗兔IgG抗体孵育，使用超敏ECL化学发光检测试剂盒进行检测；采用ImageJ 1.46r软件进行灰度值半定量分析。

## 结果

一、血小板功能分析

先证者血小板经二磷酸腺苷、胶原、肾上腺素、花生四烯酸诱导后均未见聚集，而瑞斯托霉素诱导的血小板聚集正常；先证者母亲血小板对二磷酸腺苷及肾上腺素的诱导聚集反应稍低，对其余诱聚剂反应正常；先证者父亲血小板聚集功能基本正常（图1）。

**图1:**
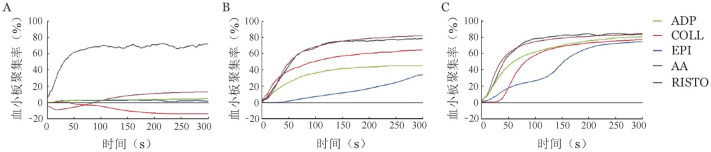
先证者（A）及其母亲（B）、父亲（C）血小板聚集曲线 **注** ADP：二磷酸腺苷；COLL：胶原；EPI：肾上腺素；AA：花生四烯酸；RISTO：瑞斯托霉素

二、流式细胞术检测血小板膜糖蛋白表达

流式细胞术检测结果显示先证者血小板表面αⅡb表达仅为0.25％，β3弱表达为9.76％，而GPⅠb表达为87.5％；先证者父母血小板表面αⅡb、β3、GPⅠb的表达基本正常（图2）。

**图2:**
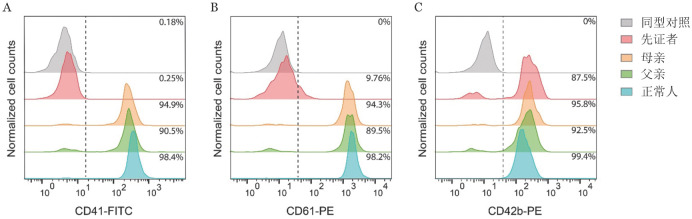
流式细胞术检测血小板膜糖蛋白表达 **注** **A** 血小板膜糖蛋白αⅡb（CD41）；**B** 血小板膜糖蛋白β3（CD61）；**C** 血小板膜糖蛋白GPⅠb（CD42b）

三、基因鉴定与分析

高通量测序结果显示先证者存在ITGA2B基因（NM_000419.5）第4外显子c.480C>G杂合错义突变与第28外显子c.2929C>T杂合无义突变。Sanger测序结果提示先证者c.480C>G突变遗传自其母亲，c.2929C>T突变遗传自其父亲，导致先证者ITGA2B基因复合杂合突变（图3）。

**图3:**
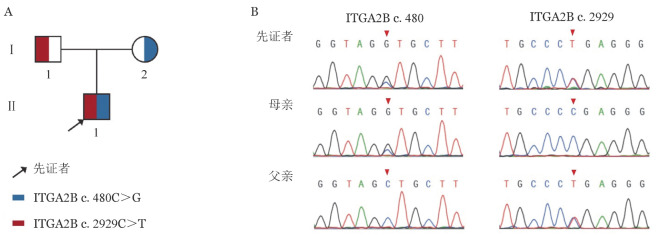
遗传性血小板无力症家系图（A）及Sanger测序结果（B）

四、mRNA剪接及水平分析

使用Mutation Taster对c.480C>G突变进行评估，结果显示为“disease causing”并提示可能存在剪接位点改变，因此我们进行了mRNA剪接分析。经RT-PCR检测，先证者及其母亲的ITGA2B基因mRNA均扩增出两种剪接体（图4A），RT-PCR产物按条带大小进行胶回收纯化后测序。结果显示正常剪接体为正常序列，异常剪接体存在c.476G-574A共99个碱基缺失，导致第4外显子发生选择性剪接（图4B、4C）。Mutation Taster对c.2929C>T突变评估为“disease causing”并提示可能存在无义介导的mRNA降解（nonsense mediated mRNA decay, NMD）。通过qRT-PCR分析该家系ITGA2B基因mRNA的相对表达水平，发现先证者及其父亲的mRNA表达水平均低于与正常对照（*P*<0.05）（图4D）。

**图4:**
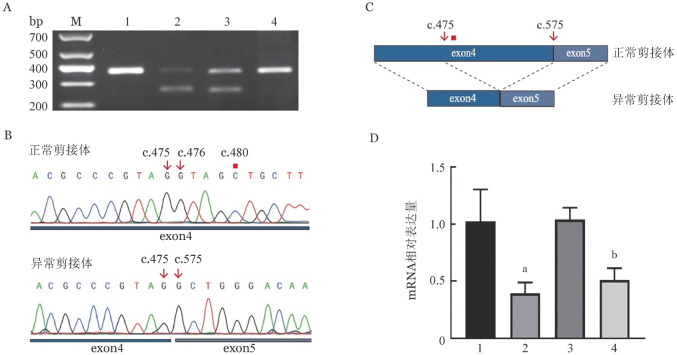
mRNA剪接与mRNA相对表达水平 **注** **A** RT-PCR扩增电泳结果（M：DNA Marker；1：正常对照；2：先证者；3：母亲；4：父亲；正常剪接体扩增片段大小为358 bp，异常剪接体扩增片段大小为259 bp）。**B** 正常及异常剪接体扩增片段Sanger测序结果。**C** 根据测序结果绘制剪接体示意图，红色正方形指示c.480位点。**D** qRT-PCR检测mRNA相对水平（1：正常对照；2：先证者；3：母亲；4：父亲。与正常对照比较，^a^ *P*<0.05）

五、生物信息学分析

为了探究c.480C>G突变导致mRNA选择性剪接的分子机制，利用ESE Finder 3.0对第4外显子区域进行剪接元件与剪接位点的预测与分析，发现c.480C>G突变并未破坏剪接因子SR蛋白结合位点，即外显子剪接增强子（exonic splicing enhancer, ESE）序列，但在SR蛋白SF2/ASF的结合位点下游产生了与其有1个碱基重叠的hnRNP A1结合位点5′-uagGug-3′（分值为4.24200），并且产生了5′剪接位点（5′SS）5′-agaagacgcccguagguagGugcuuuuugg-3′（分值为10.09250）（大写字母G表示c.480C>G突变位点）。

c.480C>G突变发生选择性剪接，使得表达产物发生33个氨基酸残基（p.S160-S192）缺失，而c.2929C>T无义突变导致第977位的精氨酸（Arg）变为终止信号，使得翻译提前终止，产生C端缺失63个氨基酸残基（p.R977-E1039）的截短型蛋白。为了明确突变型蛋白的结构变化和功能影响，构建野生型和突变型αⅡb亚基的蛋白三维结构模型。与野生型αⅡb亚基β-propeller结构域（图5A）相比，p. S160-S192缺失型蛋白的β-propeller结构域第2 blade（W2）缺失2条β链和1个α螺旋（图5B）。与野生型αⅡb亚基（图5C）相比，p.R977-E1039缺失的截短型蛋白缺失了胞质域（CD）、跨膜域（TM）以及胞外Calf-2结构域1条β链（图5D）。

**图5:**
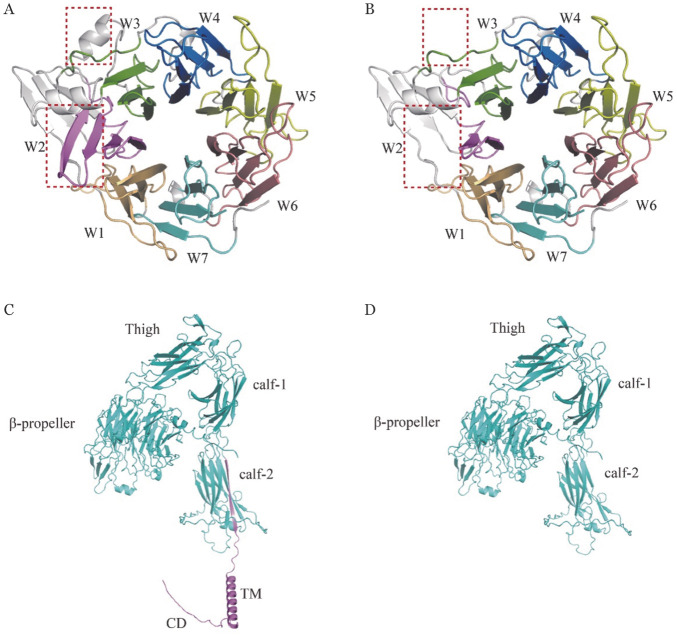
αⅡb亚基的蛋白三维结构模型 **注** **A、B** 分别为野生型和p.S160-S192缺失型αⅡb亚基β-propeller结构域三维结构模型图（β-propeller结构域包含7个“W”样blade，图中标注为W1～W7；红色虚线方框指示缺失的β链和α螺旋所对应的区域）；**C、D** 分别为野生型和p.R977-E1039缺失的截短型αⅡb亚基完整结构模型图（对结构域进行标注；紫色部位指示CD、TM及calf-2一条β链缺失所对应的区域）

六、Western blot分析蛋白表达

通过Western blot检测家系成员血小板总αⅡb、β3蛋白的表达情况，结果发现先证者血小板总蛋白中未检测到αⅡb条带，检测到β3微弱条带；先证者母亲和父亲的αⅡb和β3条带大小正常（图6A）。条带经灰度值半定量分析，结果显示先证者β3的相对表达量为正常人的11.36％，先证者母亲及父亲αⅡb、β3的表达较正常人均有不同程度的减低（*P*<0.05，图6B）。

**图6:**
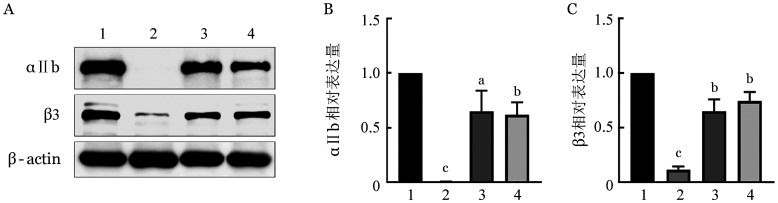
遗传性血小板无力症家系成员血小板总αⅡb、β3蛋白的表达 **注** **A** Western blot检测αⅡb、β3蛋白表达（1：正常对照；2：先证者；3：母亲；4：父亲）；**B、C** 分别为灰度值半定量分析血小板总αⅡb、β3蛋白的相对表达量（1：正常对照；2：先证者；3：母亲；4：父亲。与正常对照比较，^a^*P*<0.05，^b^*P*<0.01，^c^*P*<0.001）

## 讨论

GT由瑞士儿科医师Eduard Glanzmann于1918年首次报道，故又称Glanzmann病，是一种由ITGA2B和或ITGB3基因突变导致血小板整合素αⅡbβ3量或质的缺陷引起的血小板功能障碍性疾病[6]。αⅡbβ3仅在巨核细胞谱系中表达，是血小板表面最丰富的糖蛋白[1],[4]。αⅡbβ3为Ca^2+^依赖性异二聚体，在内质网中αⅡb前体与β3前体在Ca^2+^的参与下，以1∶1的比例组装形成αⅡbβ3复合物前体，而后才能运输到高尔基体中进行加工和修饰，形成成熟的αⅡbβ3复合物，最后通过开放管道系统定位于α颗粒和细胞膜表面[10]–[11]。未与β3亚基形成复合物的αⅡb单体或形成不稳定及成熟障碍的αⅡbβ3复合物很可能滞留在内质网中并被降解，而β3单体既可以被降解也可以与αV亚基结合形成玻连蛋白（Vitronectin, VN）受体αVβ3[12]。因此，任何影响αⅡbβ3复合物形成、成熟或干扰其细胞内运输的基因缺陷，均可导致血小板表面αⅡbβ3的表达量减少而引起遗传性GT。

αⅡb共含有1039个氨基酸残基，其结构域包含β-propeller域、Thigh域、Calf-1域、Calf-2域、跨膜域和胞质域[13]。αⅡb亚基N-末端为7个含40～60个氨基酸的同源重复序列组成的β-propeller结构域，形成7个“W”样blade，每一个blade由4条β链组成，其中第4～7 blade中包含4个Ca^2+^结合位点[12],[14]–[15]。β-propeller结构域是αⅡb最重要的结构和功能区，参与复合物的组装形成和配体的结合。

本研究中血小板聚集试验及流式细胞术提示先证者血小板表面αⅡbβ3严重缺陷，导致Ⅰ型GT表型。基因测序发现先证者及其母亲ITGA2B基因c.480C>G突变，导致第4外显子发生5′选择性剪接，产生c.476G-574A（p.S160-S192）缺失的异常剪接体。ITGA2B基因c.480C>G突变由French等[16]首次报道。国内孙安霞等[17]报告了2例患者基因型和临床表型，未阐明其致病机制。为进一步探究该突变位点发生选择性剪接的分子机制，使用ESE Finder 3.0预测结果表明，c.480C>G突变并未破坏ESE序列，但在SR蛋白SF2/ASF的结合位点下游产生了与其部分重叠的hnRNP A1蛋白结合位点5′-uagGug-3′，并且激活了5′SS剪接位点5′-agaagacgcccguagguagGugcuuuuugg-3′。研究表明，hnRNP A1是真核生物中重要的剪接因子，参与剪接调控，影响5′端剪接位点[18]。hnRNP A1的最高亲和力结合位点（winner序列）是5′-uaggga/u-3′，而含有核心基序“ag”的作用元件足以与hnRNP A1结合，其亲和力从强到弱为：uagg>uag>cagg>cag>aaag（5′→3′）[19]。所以我们认为5′-uagGug-3′是hnRNP A1蛋白的高亲和力结合位点。一般来说，外显子上ESE序列与SR蛋白家族结合产生正剪接信号，外显子剪接沉默子（exonic splicing silencer, ESS）序列与hnRNP蛋白家族结合产生负剪接信号[19]。据报道，在一些发生选择性剪接的外显子中，ESS与ESE序列重叠会导致hnRNP A1和SR蛋白对作用元件的竞争性结合[20]，hnRNP A1在5′剪接位点选择中拮抗SF2/ASF和SC35活性，导致5′剪接位点激活[21]–[22]。这与我们预测的结果相符，hnRNP A1和SR蛋白SF2/ASF的竞争性拮抗作用很可能是c.480C>G突变发生选择性剪接的分子机制。这个异常剪接体导致β-propeller结构域第2 blade缺失2条β链和1个α螺旋。有研究表明，β-propeller结构域的第1～3 blade是αⅡb与β3形成复合物的重要区域，虽然第4～7 blade含有钙结合结构域，但对于复合物的形成不是必需的[9],[23]。据报道，第1～3 blade中发生的两个小缺失（p.A106-Q111del, p.S129-S161del）、一种错义突变（p.F171C）破坏了复合物的形成[12]，所以我们推测p.S160-S192的缺失会导致αⅡbβ3复合物的形成障碍，αⅡb、β3滞留在内质网中并被降解，从而使血小板表面αⅡbβ3的表达缺如。

ITGA2B基因c.2929C>T无义突变由Giovanna D′Andrea等于2002年首次报道[24]，对患者基因型与临床表型进行分析研究，评估了患者出血严重程度，但未阐明其致病机制。本研究中，先证者及其父亲c.2929C>T无义突变，致外周血中ITG2AB基因 mRNA水平减低，说明该无义突变产生的mRNA提前终止密码子（premature termination codon, PTC），很可能触发了机体的mRNA监控机制，从而引起NMD，但其导致mRNA降解的水平及机制仍需在细胞水平中进一步研究。而未被降解的mRNA翻译成C-端缺失63个氨基酸残基（p.R977-E1039）的截短型蛋白，导致αⅡb亚基的胞质域、跨膜域以及胞外Calf-2结构域1条β链的缺失。研究表明，Calf-2结构域的破坏[25]及p.S870X的无义突变[26]并不会影响复合物的形成，但所形成的αⅡbβ3复合物稳定性降低，且向高尔基体转运障碍，在内质网中发生降解，从而阻碍血小板表面αⅡbβ3复合物的表达。所以我们认为c.2929C>T突变（p.R977-E1039缺失）不影响αⅡbβ3复合物的组装形成，但使其无法运输到血小板表面，而在内质网中发生降解。

综上所述，先证者ITGA2B基因第4外显子c.480C>G与第28外显子c.2929C>T复合杂合突变，无法产生正常的αⅡb亚基，可能导致αⅡbβ3复合物形成障碍及所形成的复合物稳定性减低或运输障碍，结合流式细胞术及Western blot检测出先证者血小板膜及血小板总的αⅡb表达缺失和β3严重降低，推测异常的αⅡb、β3在胞内已经发生降解，从而阻碍了血小板表面αⅡbβ3的表达，致先证者产生Ⅰ型GT表型。而先证者的父亲和母亲均属于单杂合突变，所以不发病。
